# A Novel Radiographic Classification of the Proximal Tibiofibular Joint

**DOI:** 10.3390/medicina62081559

**Published:** 2026-08-14

**Authors:** Berliet Assad Gomes, Lucas Pires, Rodrigo Mota Pacheco Fernandes, André Heringer Raposo, Alair Sarmet Santos, Marcus Dias, Edson Roderjan Filho, Marcio Babinski

**Affiliations:** 1 Programa de Pós-Graduação em Ciências Médicas, Hospital Universitário Antônio Pedro, Universidade Federal Fluminense, Rio de Janeiro 24033-900, Brazil; berliet@artroquadril.com.br (B.A.G.); rodrigouff@gmail.com (R.M.P.F.); edsonroderjan@gmail.com (E.R.F.); mababinski@id.uff.br (M.B.); 2 Departamento de Morfologia, Universidade Federal Fluminense, Rio de Janeiro 24210-150, Brazil; 3 Departamento de Medicina, Universidade de Vassouras, Rio de Janeiro 27700-000, Brazil; andrehraposo@gmail.com; 4 Departamento de Radiologia, Hospital Universitário Antônio Pedro, Universidade Federal Fluminense, Rio de Janeiro 24033-900, Brazil; alairsarmetsantos@gmail.com; 5 Instituto Nacional de Ortopedia e Traumatologia, Rio de Janeiro 20940-070, Brazil; mvortopedia@hotmail.com

**Keywords:** knee, tibiofibular joint, radiographic classification, fibula, morphology, morphometry, agreement

## Abstract

*Background and Objectives*: We aim to develop a simple radiographic classification for describing the morphological inclination of the proximal tibiofibular joint (PTFJ) based on composite measurements obtained from standard anteroposterior and lateral knee radiographs, and to evaluate its interobserver and intraobserver reproducibility. *Materials and Methods*: A retrospective analysis was performed on 100 standard anteroposterior and lateral knee radiographs from skeletally mature individuals. Proximal fibular articular-surface inclination was measured in both projections, and a composite index value was calculated and expressed in degrees. Based on percentile thresholds derived from reference measurements, joints were classified into three categories: low inclination (<29.4°), intermediate inclination (29.4°–35.5°), and high inclination (≥35.6°). Six observers, blinded to reference measurements, independently assessed all radiographs by performing angular measurements and assigning classifications. Data distribution was assessed using the Kolmogorov–Smirnov test. Comparisons were performed using the Chi-square, Wilcoxon, and Friedman tests. Intra- and interobserver agreement for measures were evaluated using the Intraclass Correlation Coefficient (ICC). For comparisons involving two ordinal angular classifications, both inter- and intraobserver agreement were assessed using the Weighted Cohen’s Kappa coefficient ($K_{W})$. For comparisons involving more than two ordinal angular classifications, interobserver agreement was assessed using Gwet’s AC2 coefficient (GAC2). A *p* value < 0.05 was considered significant. *Results*: The final sample comprised 59 female (59.0%) and 41 male (41.0%) radiographs. According to the proposed classification, 31% of joints were categorized as low inclination, 35% as intermediate, and 34% as high, demonstrating a balanced distribution across categories. Interobserver agreement was higher for the composite index and final classification than for isolated anteroposterior or lateral measurements, supporting the internal consistency of the system. *Conclusions*: This radiographic classification provides a simple and reproducible method for standardized assessment of proximal fibular articular-surface inclination using routine knee radiographs. The findings establish a morphologic framework with excellent measurement reliability. However, external validation in independent cohorts and correlation with clinical outcomes are required before broader clinical application or prognostic use can be established.

## 1. Introduction

The proximal tibiofibular joint (PTFJ) is classically described as a plane-type synovial articulation formed between the lateral condyle of the tibia and the head of the fibula. It is enclosed by a fibrous capsule reinforced by anterior and posterior proximal tibiofibular ligaments, contributing to its intrinsic stability [1]. Anatomical studies have demonstrated considerable variability in the size, orientation, and inclination of the articular surfaces, with the joint axis varying between transverse and oblique planes [2,3].

Several adjacent structures—most notably the fibular collateral ligament, popliteofibular ligament, and popliteus tendon—contribute to the stabilization of the posterolateral corner of the knee, a region of critical biomechanical importance, particularly in the setting of ligamentous injuries [4,5]. Additionally, the interosseous membrane, a dense fibrous sheet connecting the tibial and fibular shafts, plays a secondary role in stabilizing the PTFJ and dissipating torsional forces across the leg [6,7].

Biomechanically, fibular rotation and translation occur during ankle dorsiflexion and plantarflexion. The degree of fibular motion is influenced by the inclination of the PTFJ: a more horizontal joint allows for greater external rotation of the fibular head, whereas a more vertical orientation reduces articular congruence and increases susceptibility to subluxation or dislocation [1,6].

Despite its clinical significance in various settings—including posterolateral corner instability, tibial lengthening procedures, and chronic knee pain—morphological assessment of the PTFJ remains underutilized. Prior classification systems have substantially contributed to the understanding of PTFJ morphology. However, most were developed from cadaveric studies or relied on advanced imaging modalities such as CT and MRI, limiting their routine applicability because of higher costs, reduced availability, and, in the case of CT, radiation exposure [3,7,8].

Although previous classification systems have improved the understanding of PTFJ morphology, their routine clinical application remains limited. Most rely on cadaveric specimens or cross-sectional imaging modalities such as CT or MRI, which are associated with higher costs, limited availability, and greater radiation exposure in the case of CT. Furthermore, these classifications were primarily developed for anatomical description rather than for rapid radiographic assessment in everyday orthopedic practice. Consequently, no simple, reproducible, and widely accessible radiographic classification based on conventional plain radiographs has been established for routine clinical use.

Therefore, the aim of this study was to develop a simple, low-cost, non-invasive radiographic morphological classification of the proximal tibiofibular joint based on conventional plain radiographs and to evaluate its intraobserver and interobserver reliability.

## 2. Materials and Methods

This retrospective, cross-sectional radiographic study was approved by the Ethics Committee of the Universidade Federal Fluminense (protocol number 55407422.0.0000.5243). No informed consent was needed for this study.

### 2.1. Sample Size and Selection

This study included 100 participants. Because the study involved multiple reliability analyses, including inter-rater and intra-rater agreement, among different numbers of raters or evaluations (two, three, and six) and both continuous and ordinal outcomes, no single sample size formula was applicable to all analyses. Instead, the sample size is justified based on the precision of the reliability estimates and references. Bland and Altman (1986) provide a formula for estimating the confidence interval of the 95% limits of agreement, which corresponds to a relatively narrow confidence interval, providing adequate precision for the estimation of agreement [9]. The authors showed that a sample size of 100 can be considered adequate with methodological recommendations for agreement studies with two raters or evaluations, because for a sample size of *n* = 100, the margin of error of the confidence interval to the agreement can be approximated as ±0.34 s, less than ±0.5 s, where s represents the standard deviation of the differences between measurements. So, a sample size of 100 provides a relatively narrow confidence interval for agreement, indicating adequate precision for agreement estimation. Additionally, for continuous measurements, recommendations by Bonett (2002) [10] indicate that precision depends on the number of subjects, the number of raters (greater than or equal to 2), and the expected Intraclass Correlation Coefficient (ICC), and with 100 participants, the study provides sufficiently precise estimates of the ICC, with relatively narrow 95% confidence intervals, to the different numbers of raters or evaluations. For example, by Bonett’s formula [10], assuming an expected ICC of 0.80, a sample of 100 participants evaluated by six raters provides an expected 95% confidence interval with an approximate width of 0.11 (±0.057), supporting adequate precision for estimating both inter-rater and intra-rater reliability. For ordinal classifications, agreement was assessed using Weighted Kappa and Gwet’s AC2 coefficient, and the same sample of 100 participants ensured stable estimation of these agreement coefficients and their corresponding confidence intervals. Likewise, Gwet (2021) [11] reported that for analysis with weighted agreement coefficients, including Weighted Kappa and Gwet’s AC2, sample sizes around 100 observations generally provide reliable agreement estimates for ordinal scales with a limited number of categories. Furthermore, methodological guidelines for reliability research, like Bujang e Baharum (2017) [12], indicate that samples between 50 and 100 subjects are typically adequate for Weighted Kappa analyses involving clinical classification systems. Therefore, a sample size of 100 was considered appropriate and consistent with methodological recommendations to support all planned reliability analysis.

Hence, one hundred anonymous knee radiographs from skeletally mature individuals were selected from the radiographic archive of the Orthopedics and Traumatology Service of a university hospital between 2018 and 2021. All radiographs included anteroposterior (AP) and lateral views acquired during routine clinical assessment.

Inclusion criteria were complete knee radiographs demonstrating physeal closure of the proximal tibia and fibula, adequate image quality, and absence of rotational artifacts. Exclusion criteria included: (1) severe gonarthrosis (Kellgren–Lawrence grade III or IV); (2) osteonecrosis of the femoral condyles; (3) presence of orthopedic implants; (4) coronal angular deformities >10° in valgus or varus; and (5) any fracture, luxation and previous surgery involving the elements of the joint. Radiographic adequacy was assessed before image analysis by consensus among the three evaluators. For anteroposterior (AP) radiographs, adequate rotational positioning was defined by superimposition of the fibular head over the lateral tibial condyle by approximately one-third to one-half of the maximum diameter of the fibular head. For lateral radiographs, adequate positioning was defined by complete superimposition of the medial and lateral femoral condyles. Radiographs not meeting these criteria or presenting technical limitations that could compromise angular measurements were excluded from the analysis.

In total, 138 radiographs were evaluated with 38 exclusions (2 fracture sequelae treated with open reduction and internal fixation using plates and screws, 8 postoperative valgus-producing osteotomies stabilized with plate-and-screw constructs, 10 tricompartmental total knee arthroplasties, 10 knee radiographs showing osteoarthritis, and 8 technically inadequate radiographs due to rotational malposition). Only one knee per patient was evaluated.

### 2.2. Angle Measurements

All images were analyzed using Microsoft PowerPoint 2021 (Windows version) with integrated drawing tools. Standardized reference lines with a fixed thickness of 1 point were provided to all evaluators, who could independently reposition them according to the predefined anatomical landmarks. Observers were allowed to use zoom to improve visualization of anatomical landmarks. No brightness or contrast adjustments were performed. First, the three board-certified orthopedic surgeons involved in the study (B.G., R.F. and M.B.), all full members of the Brazilian Society of Orthopedics and Traumatology (SBOT), performed all measurements in consensus. Since only angular measurements were obtained, image calibration was not required. Measurements were recorded to one decimal place.

**Figure 1:**
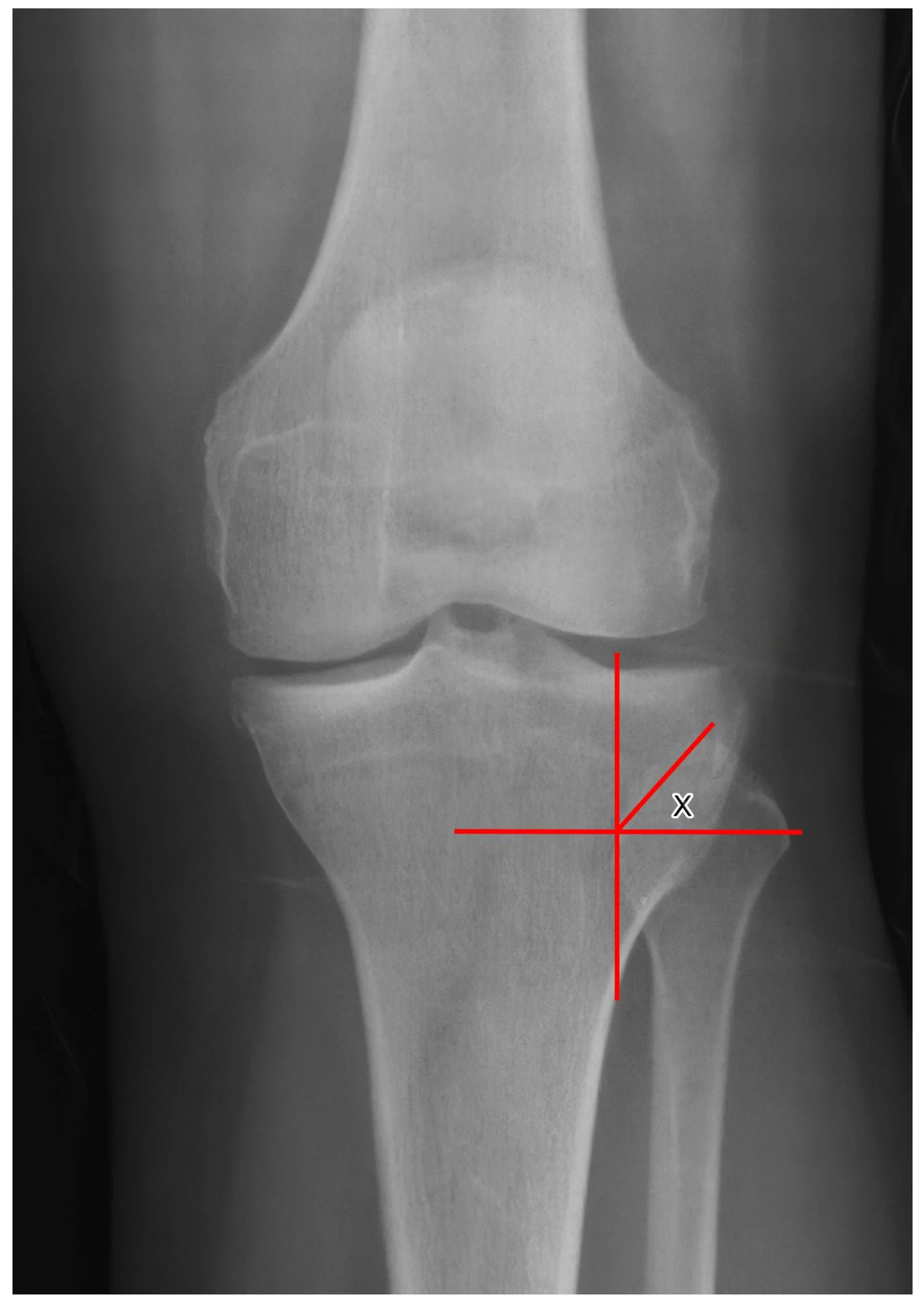
Measurement of the proximal fibular articular-surface inclination angle in the anteroposterior radiographic view. The angle was determined by constructing three reference lines: the first line was drawn tangentially along the medial cortex of the fibular diaphysis; the second line was drawn perpendicular to the first, with its intersection positioned at the most inferior portion of the proximal tibiofibular joint; and the third line originated at the apex of the fibula and was drawn tangentially along the medial surface of the fibular head. The angle between the second and third lines represents the proximal tibiofibular joint inclination angle.

**Figure 2:**
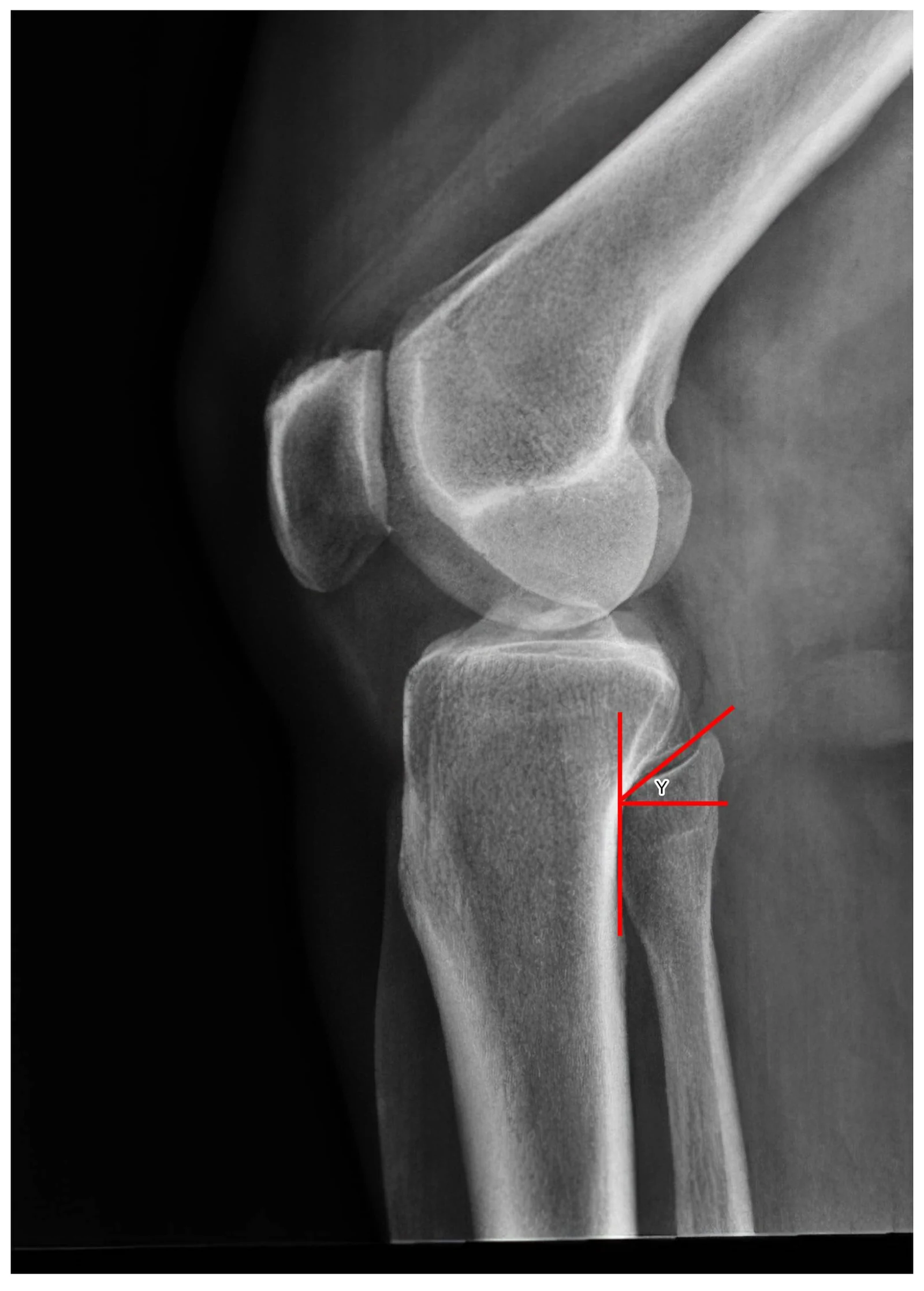
Measurement of the proximal fibular articular-surface inclination angle in the lateral radiographic view. The angle was determined by constructing three reference lines: the first line was drawn tangentially along the anterior cortex of the fibular diaphysis; the second line was drawn perpendicular to the first, with the intersection positioned at the base of the proximal tibiofibular joint; and the third line originated at the apex of the fibular styloid process and was drawn tangentially along the anterior aspect of the fibular head. The angle between the second and third lines represents the sagittal inclination angle of the proximal tibiofibular joint.

The two primary angles were defined as follows:

- Coronal (AP) angle: A reference line was drawn along the medial cortex of the fibular diaphysis. A second line perpendicular to this reference defined the horizontal plane at the base of the fibular articular surface. A third oblique line connecting the intersection point to the slope of the articular surface defined the coronal inclination angle (Figure 1).
- Lateral angle: The same procedure was applied using the anterior cortex of the fibular diaphysis as the reference line (Figure 2).
- A composite index was calculated as the arithmetic mean of the AP and lateral values.

### 2.3. Development of the PTFJ Stratification

After the angle measurements, descriptive statistics were obtained and data normality was assessed using the Kolmogorov–Smirnov test. To define the morphological categories of the PTFJ, the distribution of composite index was stratified according to the 33rd and 66th percentiles (29.4° and 35.5°, respectively). These cutoff values were distribution-based and derived from the statistical characteristics of the sample studied, with the purpose of generating balanced groups for analytical comparison. This resulted in three categories:

- Low inclination: <29.4°;
- Intermediate inclination: 29.4°–35.5°;
- High inclination: ≥35.6°.

Following definition of these cutoff values, all radiographs were classified by the same three board-certified orthopedic surgeons (B.G., R.F. and M.B.), all full members of the Brazilian Society of Orthopedics and Traumatology (SBOT), reaching consensus as well. This classification was adopted as the reference standard classification for all subsequent agreement analyses. This design was intended to assess measurement reliability rather than diagnostic accuracy or external validation in an independent cohort.

### 2.4. Independent Observer Assessment (Reliability Study)

After the development of the classification system, the entire radiographic sample was independently assessed by six new observers, all board-certified orthopedic and trauma specialists. Three observers had <5 years of experience (evaluators 1–3), while three had ≥5 years (evaluators 4–6), and they were not involved in the study whatsoever. All observers received three PowerPoint files: (1) the set of 100 radiographs with pre-drawn reference lines and a digital protractor; (2) a classification value sheet; and (3) an instructional video demonstrating the measurement protocol. Observers had 30 days to complete the first assessment and returned the measurements via Excel spreadsheet.

Then, the image set was randomized using RANDOMIZER software (https://www.randomizer.org/, accessed on 1 January 2021) and, following a 90-day interval, was resent for a second evaluation.

### 2.5. Statistical Analysis

Descriptive statistics included mean, median, standard deviation, coefficient of variation, proportions, simple frequency distributions, and cross-tabulations. Analyses were performed per observer, by experience group, and globally.

Normality was tested using the Kolmogorov–Smirnov test. Because most quantitative variables demonstrated non-normal distribution, nonparametric tests were applied: paired comparisons using the Wilcoxon signed-rank test and comparisons involving more than two paired measures using the Friedman test. Associations between qualitative variables were assessed using the Chi-square test.

Inter- and intraobserver reliability were assessed using the Intraclass Correlation Coefficient (ICC). Interobserver reliability was evaluated using a two-way random-effects ICC model, based on absolute agreement for single measurements. Intraobserver reliability was evaluated using a two-way mixed-effects ICC model, also based on absolute agreement for single measurements. For comparisons involving two ordinal angular classifications, both inter- and intraobserver agreement were assessed using the Weighted Cohen’s Kappa coefficient ($K_{w}),$ with quadratic weights. For comparisons involving more than two ordinal angular classifications, interobserver agreement was assessed using Gwet’s AC2 coefficient (GAC2), also with quadratic weights. Agreement levels were interpreted according to Weir (2005) [13]: 0.00–0.20 was poor; 0.21–0.40 was fair; 0.41–0.60 was good; 0.61–0.80 was very good; and 0.81–1.00 was excellent.

All analyses were performed using IBM SPSS Statistics v22.0 (IBM Corp., Armonk, NY, USA), RStudio/Posit (Posit Software, Boston, MA, USA, version 2024.12.1), with R version 4.4.1 (R Foundation for Statistical Computing, Vienna, Austria) and Microsoft Excel 2015 (Microsoft Corporation, Redmond, WA, USA). Statistical significance was set at *p* < 0.05.

## 3. Results

### 3.1. Sample Characteristics

The sample consisted of 59 female patients (59.0%) and 41 male patients (41.0%). Laterality distribution was asymmetric, with 67.0% being right-sided radiographs and 33.0% left-sided radiographs. Sex distribution did not differ significantly (binomial test, *p* = 0.089), whereas laterality showed a significant predominance of right-sided images (*p* = 0.001). No significant association was found between sex and laterality (Chi-square test, *p* = 0.525). The mean age was 54 ± 18 years.

### 3.2. Angle Measurements and Classification Distribution

Descriptive statistics of the measured angles are shown in Table 1. AP angles ranged from 2° to 66° (mean 43.2° ± 13.4°), lateral angles from 4° to 51° (mean 19.6° ± 8.7°), and composite indexs from 7° to 52.5° (mean 31.4° ± 8.5°). The Kolmogorov–Smirnov test demonstrated non-normal distribution for all three variables (AP: D = 0.1259 and *p* = 0.0005; lateral: D = 0.1089 and *p* = 0.0053; composite: D = 0.1118 and *p* = 0.0036). Based on this system, 31% of the sample was classified as low, 35% as intermediate, and 34% as high inclination (Table 2).

### 3.3. Classification Agreement Relative to the Reference Standard

To provide an overall estimate of classification performance and minimize the influence of temporal variability, results from both assessment rounds were pooled for frequency analysis.

Classification agreement was evaluated using the consensus reference classification established by the three primary researchers. In the first assessment, 80.2% (962 of 1200) of evaluator classifications were concordant with the consensus reference classification, whereas 13.0% were assigned to a higher category and 6.8% to a lower category. In the second assessment, concordance decreased to 64.5% (774 of 1200), with 19.2% of classifications assigned to a higher category and 16.3% to a lower category, suggesting reduced measurement consistency over time.

Across all 1200 evaluations, 72.3% of classifications were concordant with the consensus reference classification, whereas 16.1% were assigned to a higher category and 11.6% to a lower category. When stratified by experience, evaluators with less than five years of practice achieved 71.5% concordance with the consensus reference classification, with 15.7% of classifications assigned to a higher category and 12.8% to a lower category. Evaluators with five or more years of experience demonstrated 73.2% concordance, with 16.5% of classifications assigned to a higher category and 10.3% to a lower category, indicating similar overall agreement between experience groups. These data are summarized in Table 3.

### 3.4. Intraobserver Agreement

Intraobserver reliability analyses showed that, overall, no consistent systematic differences were observed between the two assessment rounds for most evaluators (Wilcoxon test, *p* > 0.05 in the majority of comparisons). Agreement levels varied according to the type of measurement and evaluator experience.

Among evaluators with five years of experience or less (evaluators 1–3), AP measurements demonstrated fair agreement (global ICC = 0.40; 95% CI: 0.30–0.49), and lateral angles also showed fair agreement (ICC = 0.44; 95% CI: 0.34–0.53). The composite index demonstrated good agreement (ICC = 0.61; 95% CI: 0.51–0.69), and the final classification also showed good agreement ($K_{w}$ = 0.64; 95% CI: 0.59–0.70).

For evaluators with more than five years of experience (evaluators 4–6), AP measurements demonstrated good agreement (ICC = 0.52; 95% CI: 0.43–0.60), while lateral angles showed fair agreement (ICC = 0.41; 95% CI: 0.31–0.50). The composite index demonstrated very good agreement (ICC = 0.73; 95% CI: 0.67–0.79), and the final classification also showed very good agreement ($K_{w}$ = 0.74; 95% CI: 0.65–0.82). When all six evaluators were analyzed together, the AP angle demonstrated fair agreement (ICC = 0.47; 95% CI: 0.40–0.53), and the lateral angle also showed fair agreement (ICC = 0.44; 95% CI: 0.38–0.50). In contrast, the composite index demonstrated very good agreement (ICC = 0.68; 95% CI: 0.62–0.72), and the final classification also demonstrated very good agreement ($K_{w}$ = 0.70; 95% CI: 0.63–0.78), representing the highest intraobserver reliability levels across measurements. Data are summarized in Table 4.

### 3.5. Agreement Between Evaluators and Reference Measurements

Agreement between individual evaluators and the reference standard (researchers’ consensus classification) varied according to the measurement type (Table 5). Across all analyses, concordance was consistently higher for the composite index and the final three-tier classification than for isolated AP and lateral measurements.

Among evaluators with five years of experience or less (evaluators 1–3), AP agreement ranged from fair to good (ICC 0.49–0.64; 95% CI 0.37–0.72), while lateral measurements demonstrated wider variability, ranging from poor to very good (ICC 0.31–0.77; 95% CI 0.17–0.83). The composite index showed good to very good agreement (ICC 0.61–0.78; 95% CI 0.49–0.83), and the final classification demonstrated good to very good concordance ($K_{w}$ = 0.65–0.81; 95% CI 0.55–0.89). For evaluators with more than five years of experience (evaluators 4–6), AP agreement ranged from fair to very good (ICC 0.43–0.76; 95% CI 0.31–0.81), while lateral agreement ranged from poor to good (ICC 0.30–0.59; 95% CI 0.17–0.67). The composite index demonstrated good to excellent agreement (ICC 0.67–0.87; 95% CI 0.56–0.90), and the final classification showed very good to excellent concordance ${(K}_{w}$ = 0.70–0.80; 95% CI 0.59–0.87).

When analyzed by experience group, less-experienced evaluators demonstrated good agreement for AP measurements (ICC = 0.54; 95% CI 0.51–0.62) and lateral measurements (ICC = 0.49; 95% CI 0.43–0.55). The composite index demonstrated very good agreement (ICC = 0.72; 95% CI 0.67–0.76), and the final classification likewise showed very good agreement ($K_{w}$ = 0.75; 95% CI 0.69–0.81). Similarly, more-experienced evaluators demonstrated good agreement for AP measurements (ICC = 0.60; 95% CI 0.55–0.65), fair agreement for lateral measurements (ICC = 0.44; 95% CI 0.37–0.50), and very good agreement for both the composite index (ICC = 0.77; 95% CI 0.72–0.80) and final classification ($K_{w}$ = 0.79; 95% CI 0.73–0.85).

Considering all evaluators together, agreement with the reference standard was good for AP measurements (ICC = 0.62; 95% CI 0.58–0.65) and lateral measurements (ICC = 0.49; 95% CI 0.45–0.53), while the composite index (ICC = 0.77; 95% CI 0.74–0.79) and final classification ($K_{w}$ = 0.80; 95% CI 0.76–0.84) demonstrated the highest concordance levels.

Wilcoxon tests only revealed statistically significant differences for isolated lateral measurements in evaluators 2 and 4 (*p* < 0.001), while most other paired comparisons did not demonstrate systematic differences.

### 3.6. Interobserver Agreement

Interobserver reliability varied according to the type of measurement and evaluator experience (Table 6). Among evaluators with less than five years of experience (evaluators 1–3), the AP angle demonstrated good agreement (ICC = 0.57; 95% CI: 0.49–0.64), with no significant differences among evaluators (Friedman test, *p* = 0.130). In contrast, the lateral angle showed fair agreement (ICC = 0.41; 95% CI: 0.32–0.50) and significant differences (*p* < 0.001), corresponding to the lowest concordance values within this group. The composite index demonstrated very good agreement (ICC = 0.78; 95% CI: 0.72–0.83), and the final classification showed good agreement (GAC2 = 0.75; 95% CI: 0.67–0.84).

For evaluators with five or more years of experience (evaluators 4–6), lower agreement levels were observed for isolated measurements. The AP angle showed fair agreement (ICC = 0.47; 95% CI: 0.38–0.55) with significant inter-evaluator differences (*p* < 0.001). The lateral angle demonstrated the lowest concordance (ICC = 0.30; 95% CI: 0.22–0.39; *p* < 0.001). In contrast, the composite index showed very good agreement (ICC = 0.72; 95% CI: 0.64–0.78), and the final classification likewise demonstrated very good agreement (GAC2 = 0.66; 95% CI: 0.60–0.75).When all six evaluators were analyzed together, the AP angle demonstrated good agreement (ICC = 0.52; 95% CI: 0.46–0.58), while the lateral angle showed fair agreement (ICC = 0.34; 95% CI: 0.28–0.41) and there were significant differences among evaluators (*p* < 0.001). In contrast, the composite index demonstrated excellent agreement (ICC = 0.85; 95% CI: 0.82–0.88), and the final classification also showed excellent agreement (GAC2 = 0.84; 95% CI: 0.80–0.88).

## 4. Discussion

The proximal tibiofibular joint (PTFJ) has historically received limited attention in routine orthopedic assessment despite its anatomical complexity and its biomechanical relevance in knee stability, ankle mechanics, and limb realignment procedures. Classical anatomical descriptions by Barnett and Napier identified three morphological types based on joint surface orientation, yet nearly one-quarter of specimens could not be adequately classified under this system [14]. Ogden later proposed a functional binary classification—horizontal versus oblique—based on articular inclination, correlating steeper orientations with reduced contact area, increased shear forces, and greater susceptibility to instability and dislocation [3,15].

Subsequent cadaveric and imaging studies confirmed the predominance of oblique morphologies in the general population [2,16], but these classifications rely primarily on CT or MRI, modalities that are not universally available and may not be justified for routine assessment.

The present study introduces a novel radiographic classification of the PTFJ based solely on standard AP and lateral knee radiographs—an accessible, low-cost, and clinically practical approach. By deriving percentile-based thresholds from objective angular measurements, we propose three inclination categories (low, intermediate, and high) that conceptually parallel Ogden’s functional system. The rationale is biomechanically supported: increasing inclination reduces the articular contact area, amplifies shear stress during axial or distraction loading, and predisposes the joint to subluxation or proximal fibular migration [7].

This has direct implications in distraction osteogenesis, where proximal fibular ascent remains a known complication [17,18]. Our classification may therefore provide a structured morphologic framework that could potentially assist in preoperative assessment. However, the present study does not establish outcome-based risk prediction, and any potential role in anticipating instability or guiding supplemental fixation should be considered hypothesis-generating pending clinical validation.

Beyond deformity correction, PTFJ morphology is increasingly relevant in ligament reconstruction, especially in procedures involving the posterolateral corner (PLC). The PTFJ is anatomically contiguous with critical stabilizing structures such as the fibular collateral ligament and popliteofibular ligament [4,5]. Variations in joint inclination may influence graft tensioning, tunnel positioning, and residual knee laxity, thereby affecting postoperative outcomes. A simple radiographic parameter that stratifies joint morphology could enhance preoperative planning in multiligament knee surgery and trauma reconstruction.

A secondary objective of this study was to evaluate the reproducibility of the proposed system, acknowledging that the reliability of any classification depends on consistency across evaluators with different levels of clinical experience. The literature is inconsistent regarding the relationship between evaluator experience and interobserver agreement [19,20,21,22]. In our analysis, less-experienced evaluators tended to assign higher AP measurement categories than the consensus reference classification, whereas more-experienced evaluators more frequently assigned lower categories; both groups showed higher values for the lateral angle than the reference measurements obtained by the researchers. These discrepancies did not translate, however, into clinically significant impairment of the classification’s performance.

Notably, the composite index demonstrated the highest reliability. Interobserver agreement was excellent for the composite index (ICC = 0.85) and for the final classification (GAC2 = 0.85). This reinforces that averaging AP and lateral measurements mitigates the instability inherent to isolated angular evaluations—particularly the lateral angle, which consistently shows the lowest interobserver agreement (ICC = 0.34). These findings echo prior studies suggesting that reproducibility is influenced less by years of practice and more by familiarity with the classification system, methodological training, image quality and even time available to conduct the classification [22,23].

Although the more-experienced evaluators demonstrated slightly better intraobserver stability, the differences between the two experience groups were small within the observer sample included in this study. Evaluator 6, from the more-experienced group, achieved the highest overall performance, whereas evaluator 1, with less than five years of experience, achieved the second-highest performance. These findings suggest that familiarity with the measurement protocol may contribute to reliability in addition to clinical experience, consistent with the observations of Shrader (2017) [24]. However, because only three evaluators were included in each experience group, no broad conclusions regarding the influence of professional experience can be drawn. Further studies including a larger and more diverse group of observers are warranted to better evaluate the relationship between clinical experience and measurement reproducibility.

Despite the lower reproducibility of the lateral angle, both the composite index and the final classification exceeded 70% agreement across 1200 measurements, supporting the robustness of the system. As Almeida (2018) [25] observed, even classifications with moderate underlying measurement variability can remain clinically useful if they offer consistent patterns that assist decision-making. The low reproducibility of the lateral angle did not compromise the reliability of the composite parameter. On the contrary, the integration of AP and lateral measurements appeared to mitigate projection-related variability and random measurement error inherent to single-plane assessment. The substantially higher ICC observed for the composite index suggests that combining orthogonal projections enhances overall measurement stability rather than amplifying isolated measurement noise.

Several authors emphasize that classification systems should be applied cautiously and with appropriate training, as interobserver variation is intrinsic to radiographic interpretation [26,27]. The present findings align with this perspective, although individual angular measurements can vary.

Overall, our findings demonstrate that isolated measurements—particularly the lateral angle—show lower interobserver reproducibility, regardless of evaluator experience. In contrast, the composite index and the final three-tier classification exhibit substantially higher reliability, reaching very good to excellent levels. These results indicate that the use of the composite angular measure and the proposed morphological classification provides a more stable and reproducible method than reliance on isolated AP or lateral angles. This suggests that single-plane angular parameters should not be interpreted independently in clinical practice, as they may be susceptible to projection variability and subjective interpretation.

The reduction in categorical concordance between the first and second assessments (80.2% to 64.5%) likely reflects small angular variations around predefined cutoff thresholds rather than substantial measurement instability. Given the 90-day interval designed to eliminate recall bias, this variation may represent natural measurement dispersion rather than true performance decline. Notably, intraobserver agreement values remained stable, supporting the overall reliability of the classification.

Beyond surgical planning, a standardized radiographic classification of PTFJ morphology may also have potential implications for rehabilitation. If future studies demonstrate associations between specific inclination patterns and joint biomechanics, instability, or functional outcomes, the proposed classification could contribute to individualized rehabilitation strategies, including exercise progression, monitoring of joint stability, and return-to-activity decisions. Furthermore, because the classification is based on conventional radiographs, it may represent a cost-effective alternative to advanced imaging for the initial morphological assessment of the PTFJ in both clinical practice and resource-limited settings. However, these potential applications remain speculative and require validation in prospective clinical studies.

This study is not without limitations. As a retrospective radiographic analysis, heterogeneity in image quality was unavoidable, despite the exclusion of inadequate studies. The large number of radiographs required substantial evaluator time, which may have contributed to reduced rigor during the second assessment. Furthermore, all evaluators were practicing orthopedic surgeons with significant clinical responsibilities, which may have influenced intraobserver consistency.

Although Microsoft PowerPoint was used for angular measurements, standardized digital calibration and consistent measurement protocols were applied across all evaluators. Dedicated radiologic software may provide additional measurement features and potentially improve absolute measurement accuracy through specialized angle measurement and image manipulation tools. However, because all observers used the same calibrated platform under identical measurement conditions, the choice of software is unlikely to have substantially influenced the interobserver and intraobserver reliability estimates, which represent the primary outcomes of this study. Nevertheless, direct comparisons between Microsoft PowerPoint and dedicated radiologic software were not performed and should be addressed in future studies to determine whether absolute angle values or measurement precision differ across platforms.

Additionally, the cutoff values (33rd and 66th percentiles) were empirically derived from the distribution of the present cohort to generate balanced groups for comparative analyses. Consequently, these thresholds should be regarded as sample-specific rather than normative and are not intended to represent definitive anatomical reference values. Their applicability to other populations remains uncertain, as the distribution of proximal tibiofibular joint inclination may differ according to demographic composition, ethnic background, imaging acquisition protocols, and other sources of population heterogeneity. Future multicenter studies including larger and more diverse cohorts are warranted to externally validate these thresholds and determine whether population-specific or universally applicable cutoff values are more appropriate. Furthermore, as the primary objective of the present study was to assess reproducibility and agreement rather than correlation between measurement planes, Bland–Altman plots were not conducted, although such analyses may be explored in future studies.

Moreover, the present study is purely morphologic and does not establish clinical or prognostic implications, as it is based on tertile stratification. Despite this, variations in proximal tibiofibular joint inclination may theoretically influence fibular rotational behavior, load transmission, and posterolateral corner stability; further investigations integrating morphologic data with biomechanical testing and surgical outcomes are necessary to determine clinically meaningful thresholds.

At present, the proposed classification should be regarded as a reproducible morphologic framework rather than a tool for direct clinical risk assessment, as external validation was not conducted. In summary, our radiographic classification provides a simple, reproducible tool for assessing proximal fibular articular-surface inclination. Future research correlating joint inclination with surgical outcomes, instability patterns, and postoperative complications will further clarify the clinical significance of PTFJ morphology.

## 5. Conclusions

The radiographic exploratory tertile-based morphological stratification proposed in this study represents a simple, accessible, and reproducible approach for assessing proximal tibiofibular joint inclination using standard knee radiographs. Although isolated AP and lateral measurements showed greater variability, the composite index and the resulting three-tier classification demonstrated excellent interobserver and intraobserver reliability.

These findings suggest that the proposed system may provide a useful preliminary framework for the standardized description of proximal fibular articular-surface inclination patterns. However, the present study was designed to evaluate measurement reproducibility rather than clinical applicability or prognostic performance. Therefore, no conclusions can be drawn regarding its clinical usefulness at this stage.

External validation in independent cohorts, together with studies correlating inclination patterns with biomechanical characteristics and clinical or surgical outcomes, will be necessary to determine the generalizability, clinical relevance, and potential role of this classification in orthopedic practice.

## Figures and Tables

**Table 1:** Descriptive statistics of the studied angles.

	Anteroposterior	Lateral	Composite
Minimum	2°	4°	7°
Maximum	66°	51°	52.5°
Mean ± SD	43.2° ± 13.4°	19.66° ± 8.7°	31.44° ± 8.5°

SD = standard deviation.

**Table 2:** Distribution of PTFJ classification by reference and evaluator experience (both assessments combined).

Classification	Reference	Evaluators < 5 Yrs (F, %)	Evaluators ≥ 5 Yrs (F, %)	All Evaluators (F, %)
Low	31 (31.0)	162 (27.0)	166 (27.7)	328 (27.3)
Intermediate	35 (35.0)	220 (36.7)	198 (33.0)	418 (34.8)
High	34 (34.0)	218 (36.3)	236 (39.3)	454 (37.8)

Frequencies (F) correspond to the sum of classifications from both assessments for each evaluator group. Researchers’ values represent the reference standard. Each experience group contributed 600 classifications (3 observers × 100 radiographs × 2 assessment rounds), totaling 1200 classifications.

**Table 3:** Cross-tabulation between reference standard classification and evaluator classification stratified by experience level.

Evaluators with < 5 Years of Experience (n = 600)				
Evaluator classification	Low	Intermediate	High	Total
Low	121 (20.2%)	26 (4.3%)	17 (2.8%)	164 (27.3%)
Intermediate	32 (5.3%)	155 (25.8%)	34 (5.7%)	221 (36.8%)
High	33 (5.5%)	29 (4.8%)	153 (25.5%)	215 (35.8%)
Total	186 (31.0%)	210 (35.0%)	204 (34.0%)	600 (100%)
**Evaluators with ≥5 Years of Experience (n = 600)**				
Evaluator classification	Low	Intermediate	High	Total
Low	135 (22.5%)	17 (2.8%)	14 (2.3%)	166 (27.7%)
Intermediate	22 (3.7%)	145 (24.2%)	31 (5.2%)	198 (33.0%)
High	29 (4.8%)	48 (8.0%)	159 (26.5%)	236 (39.3%)
Total	186 (31.0%)	210 (35.0%)	204 (34.0%)	600 (100%)

Each experience group contributed 600 classifications (3 observers × 100 radiographs × 2 assessment rounds), totaling 1200 classifications.

**Table 4:** Intraobserver agreement for angle measurements and classification.

Evaluator	AP Angle ICC	Lateral Angle ICC	Composite Index ICC	Classification $K_{w}$
1	0.49 [0.32–0.63] (Fair)	0.52 [0.36–0.65] (Fair)	0.67 [0.50–0.78] (Good)	0.72 [0.01–0.83] (Very Good)
2	0.44 [0.26–0.58] (Fair)	0.42 [0.24–0.57] (Fair)	0.59 [0.24–0.79] (Fair)	0.69 [0.50–0.88] (Good)
3	0.30 [0.11–0.47] (Poor)	0.26 [0.06–0.43] (Poor)	0.56 [0.35–0.71] (Fair)	0.55 [0.40–0.71] (Fair)
Global < 5 yrs	0.40 [0.30–0.49] (Fair)	0.44 [0.34–0.53] (Fair)	0.61 [0.51–0.69] (Good)	0.64 [0.59–0.70] (Good)
4	0.34 [0.16–0.50] (Poor) *	0.24 [0.05–0.41] (Poor) *	0.61 [0.05–0.41] (Good)	0.80 [0.68–0.92] (Very Good)
5	0.54 [0.39–0.67] (Fair)	0.36 [0.18–0.52] (Poor)	0.65 [0.48–0.77] (Good)	0.65 [0.50–0.79] (Good)
6	0.67 [0.55–0.77] (Good)	0.53 [0.37–0.66] (Fair)	0.83 [0.75–0.89] (Very Good)	0.77 [0.66–0.87] (Very Good)
Global ≥ 5 yrs	0.52 [0.43–0.60] (Good)	0.41 [0.31–0.50] (Fair)	0.73 [0.67–0.79] (Very Good)	0.74 [0.65–0.82] (Very Good)
Overall	0.47 [0.40–0.53] (Fair)	0.44 [0.38–0.50] (Fair)	0.68 [0.62–0.72] (Very Good)	0.70 [0.63–0.78] (Very Good)

ICC = Intraclass Correlation Coefficient; $K_{W}:$ Weighted Kappa Coefficient, AP = anteroposterior angle; yrs = years; * = Wilcoxon’s test < 0.05. The 95%CI can be seen between square brackets.

**Table 5:** Interobserver agreement between each evaluator and the reference standard.

Evaluator	AP Angle ICC	Lateral Angle ICC	Composite Index ICC	Classification $K_{w}$
1	0.64 [0.55–0.72] (Good)	0.77 [0.70–0.83] (Very Good)	0.78 [0.70–0.83] (Very Good)	0.81 [0.73–0.89] (Very Good)
2	0.49 [0.37–0.59] (Fair)	0.31 [0.17–0.43] (Poor) *	0.61 [0.49–0.71] (Good)	0.65 [0.55–0.75] (Good)
3	0.57 [0.47–0.66] (Good)	0.58 [0.48–0.66] (Good)	0.75 [0.68–0.81] (Very Good)	0.72 [0.65–0.80] (Very Good)
Global < 5 yrs	0.57 [0.51–0.62] (Good)	0.49 [0.43–0.55] (Good)	0.72 [0.67–0.76] (Very Good)	0.75 [0.69–0.81] (Very Good)
4	0.43 [0.31–0.54] (Fair)	0.30 [0.17–0.42] (Poor) *	0.67 [0.56–0.75] (Good)	0.70 [0.59–0.80] (Very Good)
5	0.61 [0.52–0.69] (Good)	0.59 [0.49–0.67] (Good)	0.78 [0.71–0.83] (Very Good)	0.70 [0.62–0.78] (Very Good)
6	0.76 [0.70–0.81] (Very Good)	0.58 [0.48–0.66] (Good)	0.87 [0.83–0.90] (Excellent)	0.84 [0.81–0.87] (Excellent)
Global ≥ 5 yrs	0.60 [0.55–0.65] (Good)	0.44 [0.37–0.50] (Fair) *	0.77 [0.72–0.80] (Very Good)	0.79 [0.73–0.51] (Very Good)
Overall	0.62 [0.58–0.65] (Good)	0.49 [0.45–0.53] (Good)	0.77 [0.74–0.79] (Very Good)	0.80 [0.76–0.84] (Very Good)

ICC = Intraclass Correlation Coefficient; $K_{w}$: Weighted Kappa Coefficient; AP = anteroposterior angle; * = Wilcoxon’s test value was <0.001. The 95%CI can be seen between square brackets.

**Table 6:** Interobserver agreement (ICC) for angular measurements and classification of the proximal tibiofibular joint.

Evaluators	Variable	Friedman Test	ICC (95% CI)	Agreement
Evaluators 1–3 (<5 years)	AP angle	0.130	0.57 (0.49–0.64)	Good
	Lateral angle	<0.001	0.41 (0.32–0.50)	Fair
	Composite index	0.051	0.78 (0.72–0.83)	Very good
	Classification	0.051	0.75 (0.67–0.84) *	Good
Evaluators 4–6 (≥5 years)	AP angle	<0.001	0.47 (0.38–0.55)	Fair
	Lateral angle	<0.001	0.30 (0.22–0.39)	Fair
	Composite index	0.056	0.66 (0.60–0.72)	Very good
	Classification	0.055	0.69 (0.61–0.76) *	Very good
All evaluators (n = 6)	AP angle	0.051	0.52 (0.46–0.58)	Good
	Lateral angle	<0.001	0.34 (0.28–0.41)	Fair
	Composite index	0.051	0.85 (0.82–0.88)	Excellent
	Classification	0.051	0.85 (0.81–0.90) *	Excellent

CI = confidence interval. * Agreement performed by GAC2: Gwet’s AC2 coefficient.

## Data Availability

The datasets used and/or analyzed during the current study are available from the corresponding author on request.

## Abbreviations

The following abbreviation is used in this manuscript:

PTFJ	Proximal tibiofibular joint
